# *Giardia duodenalis* in Ugandan Children Aged 9–36 Months in Kampala, Uganda: Prevalence and Associated Factors

**DOI:** 10.4269/ajtmh.22-0436

**Published:** 2023-05-30

**Authors:** Grace Ndeezi, Siobhan M. Mor, Luke R. Ascolillo, Hannington B. Tasimwa, Ritah Nakato, Lilian N. Kayondo, Saul Tzipori, David Mukunya, Jeffrey K. Griffiths, James K. Tumwine

**Affiliations:** ^1^ Department of Paediatrics and Child Health, School of Medicine, Makerere University College of Health Sciences, Kampala, Uganda;; ^2^ Institute for Infection, Veterinary and Ecological Sciences, University of Liverpool, Liverpool, United Kingdom;; ^3^ International Livestock Research Institute, Addis Ababa, Ethiopia;; ^4^ Department of Public Health and Community Medicine, Tufts University School of Medicine, Boston, Massachusetts;; ^5^ Department of Microbiology, School of Biomedical Sciences, Makerere University College of Health Sciences, Kampala, Uganda;; ^6^ Department of Infectious Disease and Global Health, Tufts University Cummings School of Veterinary Medicine, North Grafton, Massachusetts;; ^7^ Department of Community and Public Health, Busitema University Faculty of Health Sciences, Mbale, Uganda;; ^8^ Division of Geographic Medicine and Infectious Diseases, Tufts Medical Center, Boston, Massachusetts;; ^9^ Department of Pediatrics and Child Health, Kabale University School of Medicine, Kabale, Uganda

## Abstract

*Giardia duodenalis* is a common gastrointestinal pathogen globally that has been associated with growth failure in children. Most of the studies have been done in school-age children, and there is a paucity of data in pre-school children. We determined the prevalence and factors associated with *G. duodenalis* infection in children aged 9–36 months presenting to Mulago Hospital with diarrhea or cough. Demographic and socio-economic characteristics, animal ownership, medical history, and physical examination findings were recorded. Stool was tested for *G. duodenalis* using real-time quantitative polymerase chain reaction (qPCR), and additional tests included stool microscopy and qPCR for *Cryptosporidium*. The overall prevalence of *G. duodenalis* infection was 6.7% (214/3,173). In children with diarrhea the prevalence was 6.9% (133/1,923), whereas it was 6.5% (81/1,250) in those with cough as the main symptom. Of 214 children with *G. duodenalis* infection, 19 (8.9%) were co-infected with *Cryptosporidium*. Older children (25–36 months) were more likely to have *G. duodenalis* infection (adjusted odds ratio [aOR]: 2.93, 95% CI: 1.93–4.43). Use of an unimproved toilet (aOR: 1.38, 95% CI: 1.04–1.83) and the wet season (aOR: 1.33, 95% CI: 1.00–1.77) were associated with increased infection. Other factors associated with infection were recurrent diarrhea (aOR: 2.46, 95% CI: 1.64–3.70) and passing of mucoid stool (aOR: 2.25, 95% CI: 1.08–4.66). Having a ruminant at the homestead was also associated with infection (aOR: 1.83, 95% CI: 1.20–2.79). *Giardia duodenalis* infection occurred in 1 of 15 children aged 9–36 months with diarrhea or cough in Kampala, Uganda. Further studies are needed to clarify the zoonotic significance of *G. duodenalis* infection in this setting.

## INTRODUCTION

*Giardia duodenalis* is a common gastrointestinal pathogen in humans and is present worldwide. The distribution tends to vary by geographical location, and it is more common in low-income countries.^1^^,^^2^ *Giardia duodenalis* is also a common enteric parasite of animals, including livestock, companion animals, and wildlife, and therefore it is considered a potential zoonotic disease.^3^^–^^5^ However, evidence suggests that most cases in humans are caused by assemblages A and B, whereas animals, including most livestock species, tend to be infected with host-adapted assemblages that are rarely found in humans.^6^ *Giardia duodenalis* (syn. *G. lamblia* and *G. intestinalis*) is transmitted by contaminated drinking water or food as well as by fomites or direct physical contact with infected hosts.^7^ The infective stage of the parasite, the cyst, is encysted when released into the feces and is immediately infectious.^8^ Cysts remain infectious for months in cool, damp areas, but their survival is reduced at temperatures above 20°C, especially in tropical regions.^9^^,^^10^

*Giardia duodenalis* is three times more common in children than adults.^11^ High infection rates have also been reported in HIV-infected patients, especially before highly active antiretroviral therapy was introduced.^12^^–^^14^ Studies in low-income countries show that poor socio-economic status, hygiene, nutritional status, and immunity are risk factors for *G. duodenalis*.^15^

Studies from East African countries have shown a wide variation in prevalence and geographical distribution of *G. duodenalis*, ranging from 2.9% to 87%.^16^^–^^20^ Even within the same country and across different age groups, the prevalence and risk factors tend to vary. Few studies have described the magnitude *of G. duodenalis* in pre–school-aged children. The objective of the current study was to describe the prevalence of *G. duodenalis* and associated factors in children aged 9–36 months presenting with diarrhea or cough as key symptoms at a tertiary hospital in Uganda.

## MATERIALS AND METHODS

### Study design.

This was a cross-sectional analytical study conducted between 2014 and 2017 among children presenting with diarrhea or cough as the main symptoms to the pediatric emergency care unit of Mulago National Referral and Teaching Hospital. This was a sub-study of a hospital and household study to describe the transmission of respiratory cryptosporidiosis in children with diarrhea or cough at Mulago Hospital. In the main study, children with diarrhea or cough were tested for *Cryptosporidium*, *G. duodenalis*, and other gastrointestinal pathogens. They were followed through household visits in the community to determine if household members also had *Cryptosporidium* by stool examination. This paper reports the outcomes of *G. duodenalis* testing in the hospital setting.

### Setting.

Mulago National Referral and Teaching Hospital is situated in the capital city of Uganda and serves as a primary health care facility for people living within the surrounding areas; the majority of patients are referred from the lower heath facilities in Kampala and surrounding districts. More than half of the children admitted at the pediatric emergency unit (acute care unit [ACU]) come from the urban and peri-urban areas of Kampala. The ACU on average receives 80 patients per day, the majority of whom are aged < 5 years; the 9–36 months age group constitutes about 40% of the patient demographic. Children with diarrhea constitute 10%, whereas those with cough are approximately 25%.

### Procedure.

Children aged 9–36 months presenting with diarrhea or cough were screened for the study. Informed consent was obtained by the study doctor after explaining the details of the study and its significance to the parent or caregiver. Those who were not willing to wait for completion of the study procedures or those who could not produce a stool sample were excluded. Those who fulfilled the inclusion criteria were consecutively enrolled until the required sample size was accrued. Both inpatients and outpatients were enrolled.

Socio-demographic information, such as the child’s age and sex and the caregivers’ age and education, were obtained using a structured questionnaire. Additional household information included source of water, type of toilet facility, and presence of domestic animals or poultry on the compound. Improved water source was defined as a household that used piped water or a borehole. Improved toilet was defined as a flush toilet, ventilated improved pit latrine, or pit latrine with a slab.

Subsequently a detailed medical history and physical examination were performed on the child, which included weight, length and height, and mid-arm circumference as well as a detailed general and systemic examination. Consistency and color of the stool was determined based on inspecting the stool whenever the child passed stool or at the time of sample collection. Persistent diarrhea was defined as passing of watery or loose stool three or more times in 24 hours for a period of 14 days or more. Fever was assessed by history and confirmed by measuring the temperature. Any child who had an axillary temperature of ≥ 37.5°C was regarded as febrile. Hydration status was determined using the Integrated Management of Childhood Illness guidelines,^21^ which consider the presence or absence of sunken eyes, the sensorium, whether the skin pinch goes back very slowly or slowly, and whether a child drinks poorly or eagerly (WHO). Weight was measured with minimum clothing and no shoes using a digital two-in-one SECA (874) scale in kilograms to the nearest 0.1 kg. Measurement of length was done in a horizontal position with a wooden stadiometer for children under 2 years of age. Height was measured for children above 2 years to the nearest 0.1 cm. Stunting was defined as length or height for age ≤ −2 SD below the WHO’s Child Growth Standards median.^22^

### Stool collection and laboratory procedures.

Stool was collected from diapers or directly from the rectum using a tube connected to a syringe for those children who had diarrhea and placed into plastic containers. Samples were delivered to the laboratory within 4 hours of collection. *Giardia duodenalis* DNA from stool was extracted using the QIAamp Fast DNA Stool Mini Kit (QIAGEN, Hilden, Germany). The kit protocol was followed according to the manufacturer’s instructions except for the following modifications. After the second wash, buffer was added (AW2), and the collection tubes were centrifuged for 4 minutes to increase DNA yield. Real-time PCR was used to determine the presence of *G. duodenalis* as previously prescribed.^23^

### Statistical considerations.

The sample size was dependent on the size of the parent study. Of the 3,180 participants enrolled in the parent study, 3,173 were examined for *G. duodenalis* in the stool. This sample size resulted in an absolute precision of 0.5–1.7% (i.e., the difference between the point estimate and the 95% CI for prevalence values ranging from 2% to 50%, a precision we deemed adequate).

Data were analyzed using Stata version 14.2 (StataCorp LLC, College Station, TX). We summarized continuous variables using means with standard deviations or medians with interquartile ranges and categorical variables using their frequencies and percentages. The prevalence was defined as the proportion of participants enrolled in the study and who had a stool examined who tested positive for *G. duodenalis.* Multivariable binary logistic regression analyses was carried out to assess for the strength of association between selected exposures and *G. duodenalis* colonization. In our final model, we included variables whose *P* value was < 0.20 in bivariable analysis or where important sociodemographic characteristics or had biological plausibility. Strength of association was determined using adjusted odds ratios and 95% CIs.

## RESULTS

### Prevalence and seasonality of *G. duodenalis* infection.

**Figure 1.**
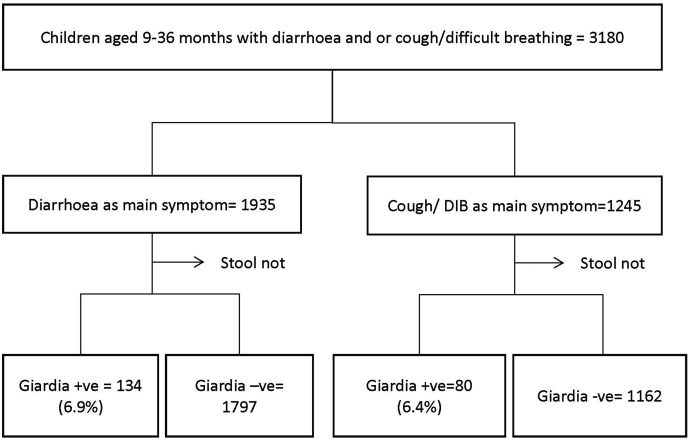
Study profile of children with *Giardia duodenalis* infection in a subset of children aged 9–36 months presenting with diarrhea at Mulago Hospital, Uganda.

**Figure 2.**
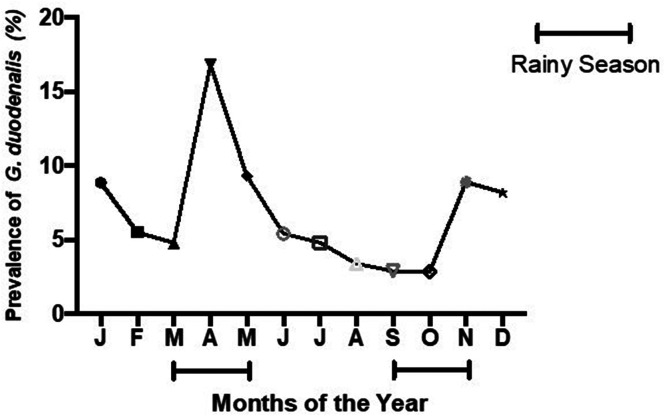
Prevalence of *Giardia duodenalis* in Ugandan children aged 9–36 months during different months of the year.

The overall prevalence of *G. duodenalis* infection was 6.7% (214/3,173). In children whose main symptom was diarrhea, the prevalence was 6.9% (133/1,923), whereas in children with cough as the key symptom it was 6.5% (81/1,250) (Figure 1). Of the 214 children with *G. duodenalis* infection, 19 (8.9%) were co-infected with *Cryptosporidium* spp. When plotted against months of the year, the frequency of *G. duodenalis* infection was higher during the wet season than during the dry season (Figure 2).

### Factors associated *G. duodenalis* infection.

The socio-demographic and household characteristics of children with and without *G. duodenalis a* are shown in Table 1. Almost half the children were aged 13–24 months (1,564/3,173; 49.3%), and only 411/3,173 (13.0%) were aged 25–36 months. Of the 214 children who tested positive for *G. duodenalis*, 112 (52.3%) were aged 13–24 months, 54 (25.2%) were 9–12 months, and 48 (22.4%) were 25–36 months old. The median age (interquartile range [IQR]) of the children who had *G. duodenalis* infection was 17 months (IQR: 12–23), compared with 14 months (IQR: 11–19) among those who were negative (*P* < 0.001). The age, level of education, and other socio-demographic characteristics of the caretakers were similar whether the child had *G. duodenalis* infection or not. The main source of water was a public tap or a stand pipe in 177/214 (82.7%) among those who had *G. duodenalis* compared with 2,223/2,959 (75.1%) without infection. Very few families had a privately connected piped water or a borehole. Most households used a toilet (improved or unimproved), commonly the pit latrine. Most of the households had animals in the compound; these were mainly domestic animals such as goats, sheep, cows, pigs, dogs, or cats.

**Table 1:** Demographic and household characteristics of children aged 6–36 months presenting with diarrhea or cough at Mulago Hospital, Uganda, by *Giardia duodenalis* infection status (*N* = 3,173)

Characteristic	Giardia positive (*n* = 214)	Giardia negative (*n* = 2,959)	*P* value
Age in months			
Median (IQR)	17 (12–23)	14 (11–19)	< 0.001
Age category, *n* (%)			
9–12 months	54 (25.2)	1,144 (38.7)	< 0.001
13–24 months	112 (52.3)	1,452 (49.1)	
25–36 months	48 (22.4)	363 (12.3)	
Sex, *n* (%)			
Male	123 (57.5)	1,641 (55.5)	0.566
Female	91 (42.5)	1,318 (44.5)	
Age of caregiver in years			
Median (IQR)	26 (23–30)	25 (23–30)	0.606
Education of caregiver, *n* (%)			
None (0–3 years)	13 (6.1)	210 (7.1)	0.786
Primary (4–7 years)	68 (31.8)	875 (29.6)	
Secondary (8–13 years)	75 (35.1)	1,108 (37.5)	
Tertiary (≥ 14 years)	58 (27.1)	763 (25.8)	
District of residence*, n* (%)			
Kampala	168 (78.5)	2,107 (71.2)	0.066
Wakiso	37 (17.3)	657 (22.2)	
Other	9 (4.2)	195 (6.6)	
Main source of water, *n* (%)			
Private connection	7 (3.3)	183 (6.2)	0.045
Public tap/standpipe	177 (82.7)	2,223 (75.1)	
Bore hole	11 (5.1)	192 (6.5)	
Protected well/spring	6 (2.8)	198 (6.7)	
Other	13 (6.1)	163 (5.5)	
Toilet improved			
No facility	0 (0.0)	5 (0.2)	0.072
Improved toilet	105 (49.1)	1,679 (56.7)	
Unimproved toilet	109 (50.9)	1,275 (43.1)	
Ruminant (goat, sheep, cow), *n* (%)			
Absent	171 (79.9)	2,465 (83.3)	0.200
Present	43 (20.1)	494 (16.7)	
Any animals, *n* (%)			
Absent	101 (47.2)	1,451 (49.0)	0.603
Present	113 (52.8)	1,508 (51.0)	
Season, *n* (%)			
Wet	130 (60.8)	1,559 (52.7)	0.022
Dry	84 (39.3)	1,400 (47.3)	
Wasting			
Present	39 (18.6)	644 (22.4)	0.203
Not present	171 (81.4)	2,238 (77.7)	
Stunting, *n* (%)			
Present	42 (19.6)	592 (20.2)	0.833
Not present	172 (80.4)	2,335 (79.8)	
Fever, *n* (%)			
Yes	145 (67.8)	2,158 (72.9)	0.101
No	69 (32.2)	801 (27.1)	
Stool *Cryptosporidium*, *n* (%)			
Yes	19 (8.9)	313 (10.6)	0.433
No	195 (91.1)	2,646 (89.4)	
Key symptom, *n* (%)			
Cough	81 (37.9)	1,169 (39.5)	0.632
Diarrhea	133 (62.2)	1,790 (60.5)	

Results of univariable and multivariable regression exploring factors in the total study population and in the subset with diarrhea as the key symptom are shown in Tables 2 and 3, respectively. Overall, factors that remained independently associated with *G. duodenalis* in multivariable analysis were older age (adjusted odds ratio [aOR]: 1.72, 95% CI: 1.23–2.41 in children aged 13–24 months; aOR: 2.93, 95% CI: 1.93–4.43 in children aged 25–36 months), using an unimproved toilet (aOR: 1.38, 95% CI: 1.04–1.83), and wet season (aOR: 1.33, 95% CI: 1.00–1.77). Among children with diarrhea as the key symptom, age and seasonality remained independently associated with *G. duodenalis* infection. Other factors were recurrent diarrhea (aOR: 2.46, 95% CI: 1.64–3.70), passing mucoid stool (aOR: 2.25, 95% CI: 1.08–4.66), and having a ruminant (goat, sheep, or cow) in the compound (aOR: 1.83, 95% CI: 1.20–2.79).

**Table 2:** Results of univariable and multivariable analysis investigating factors associated with *Giardia duodenalis* infection in children aged 9–36 months at Mulago Hospital, Uganda (*N* = 3,173)

Characteristic	OR* (95% CI)	*P* value	aOR* (95% CI)
Age category			
9–12 months	1	–	1
13–24 months	1.63 (1.17–2.28)	0.004	1.72 (1.23–2.41)
25–36 months	2.80 (1.87–4.21)	< 0.001	2.93 (1.93–4.43)
Ruminant in home (goat, sheep, cow)			
No	1	–	1
Yes	1.25 (0.89–1.78)	0.201	1.29 (0.91–1.84)
Stunting			
None	1	–	1
Present	1.04 (0.73–1.47)	0.833	0.83 (0.58–1.19)
Tertiary education			
None	1	–	1
Primary	1.26 (0.68–2.32)	0.466	1.27 (0.69–2.35)
Secondary	1.09 (0.60–2.01)	0.773	1.15 (0.62–2.12)
Tertiary	1.23 (0.66–2.28)	0.517	1.29 (0.69–2.43)
Safe water			
Safe	1	–	1
Unsafe	1.43 (0.88–2.31)	0.150	0.66 (0.41–1.08)
Improved toilet			
Improved	1	–	1
Unimproved	1.37 (1.04–1.81)	0.026	1.38 (1.04–1.83)
Season			
Dry	1	–	1
Wet	1.39 (1.05–1.85)	0.023	1.33 (1.00–1.77)
Fever			
No	1	–	1
Yes	0.78 (0.58–1.05)	0.102	0.78 (0.57–1.05)
Child sex			
Male	1	–	1
Female	0.92 (0.70–1.22)	0.566	0.89 (0.67–1.18)
Stool *Cryptosporidium*			
Negative	1	–	1
Positive	0.82 (0.51–1.34)	0.434	0.91 (0.56–1.48)

aOR = adjusted odds ratio; OR = odds ratio.

* aOR > 1 is associated with a higher odds of outcome, aOR < 1 is associated with a lower odds of outcome, and aOR = 1 means absence of an association.

**Table 3:** Results of univariable and multivariable analysis investigating factors associated with *Giardia duodenalis* infection in subset of children aged 9–36 months presenting with diarrhea at Mulago Hospital, Uganda

Characteristic	OR* (95% CI)	*P* value	aOR* (95% CI)
Age category			
9–12 months	1	–	1
13–24 months	1.18 (0.80–1.75)	0.407	1.29 (0.86–1.95)
25–36 months	2.66 (1.59–4.47)	< 0.001	3.12 (1.80–5.41)
Recurrent diarrhea			
No	1	–	1
Yes	2.46 (1.69–3.59)	< 0.001	2.46 (1.64–3.70)
Consistency of diarrhea			
Loose	1	–	1
Watery	1.42 (0.69–2.92)	0.335	1.42 (0.68–2.95)
Mucoid	2.39 (1.17–4.88)	0.017	2.25 (1.08–4.66)
Ruminant in home (goat, sheep, cow)			
No	1	–	1
Yes	1.72 (1.14–2.59)	0.009	1.83 (1.20–2.79)
Stunting			
None	1	–	1
Present	0.94 (0.60–1.46)	0.784	0.86 (0.54–1.37)
Caregiver education			
None	1	–	1
Primary	1.87 (0.78–4.47)	0.160	2.12 (0.87–5.17)
Secondary	1.62 (0.68–3.84)	0.277	1.65 (0.68–4.00)
Tertiary	1.29 (0.52–3.19)	0.578	1.58 (0.62–4.01)
Safe water			
Yes	1	–	1
No	0.63 (0.33–1.22)	0.174	0.60 (0.31–1.19)
Improved toilet			
Improved	1	–	1
Unimproved	1.49 (1.05–2.12)	0.027	1.13 (0.77–1.66)
Season			
Dry	1	–	1
Wet	1.47 (1.02–2.11)	0.038	1.46 (1.00–2.12)
Fever			
No	1	–	1
Yes	1.41 (0.97–2.05)	0.070	0.71 (0.48–1.04)
Child sex			
Male	1	–	1
Female	1.13 (0.79–1.61)	0.506	1.10 (0.76–1.56)
Stool *Cryptosporidium*			
Negative	1	–	1
Positive	0.81 (0.47–1.41)	0.454	0.87 (0.49–1.54)

aOR = adjusted odds ratio; OR = odds ratio.

* aOR > 1 is associated with a higher odds of outcome, aOR < 1 is associated with a lower odds of outcome, and aOR = 1 means absence of an association.

## DISCUSSION

This study describes the prevalence of *G. duodenalis* infection in young children aged 9–36 months presenting with diarrhea or cough/difficult breathing at a tertiary hospital in Uganda. The overall prevalence of *G. duodenalis* in these pre-school children was 6.7%, with no significant difference in the prevalence between those who had diarrhea (6.9%) and those who had cough (6.5%) as key symptoms. This is lower than what was reported in a study of asymptomatic children aged 0–12 years living in Mulago village, which is adjacent to the current study site (a prevalence of 20.1%),^17^ but similar to what was reported in a study in Dar es Salaam in children under 24 months of age.^24^ Nonetheless, our findings are consistent with other studies regarding increasing prevalence above 1 year of age. Children aged 13–24 months had the highest prevalence of *G. duodenalis* in this study, similar to what was previously reported in a multi-country study of 2,089 children.^15^ A study in Savador in Brazil among children aged 6–45 months also showed increasing prevalence of *G. duodenalis* infection with age.^25^ The low prevalence under 1 year of age could be explained by the protective effect of breastfeeding, whereas weaning has been associated with increasing infection with pathogenic parasites, including *G. duodenalis*.^26^ Whereas exclusive breastfeeding, higher socioeconomic status, and recent metronidazole use have been reported to be protective against *G. duodenalis* by other researchers,^15^ our study did not find any association with education, which is an important socioeconomic indicator. We did not assess metronidazole use in the current study, and all our study children were no longer exclusively breastfed.

The lack of difference in the prevalence between diarrhea and cough/pneumonia patients may indicate the background epidemiology of the disease in the general population of children aged 9–36 months and not necessarily symptoms of infection. Some authors have suggested that *G. duodenalis* infection is asymptomatic in high-transmission areas and may even be protective against pathogens that cause acute diarrhea.^27^^–^^29^ The symptoms of *G. duodenalis* infection have been associated with the different genotypes, with some causing diarrhea and others not.^30^ Nonetheless, in the subset of children who presented with diarrhea, *G. duodenalis* infection was more likely to be associated with recurrent diarrhea and production of mucoid stool. Other studies have also reported recurrent or relapsing diarrhea in children with *G. duodenalis* infection.^28^^,^^31^ Although *G. duodenalis* infection has been associated with growth failure in young children in a number of studies,^32^^–^^34^ this was not the case in our study. The failure to demonstrate an association could be related to the study design. Some of the studies that have shown an association were either prospective or case-control studies,^25^^,^^34^ where it was possible to demonstrate a cause-effect relationship.

In this study we found that children who came from households that had a ruminant on the compound were more likely to be infected with *G. duodenalis*. As mentioned, *G. duodenalis* is a common gastrointestinal pathogen that affects a broad range of hosts, including humans, livestock, and wildlife, and zoonotic transmission has been documented to occur because of similarities in assemblages in some species.^35^ Nonetheless, the two assemblages that are most common in humans (namely A and B) are uncommon in cattle, sheep, and goats.^6^ Although it is plausible that the feces of ruminants at the homesteads contaminated the water and food sources, facilitating transmission via the fecal–oral route, without further typing of isolates it is difficult to definitively conclude that zoonotic transmission is occurring in this setting. It is also possible that direct transmission from person to person was responsible for infection, particularly in children whose siblings were attending day care centers and schools.^36^ Transmission of *G. duodenalis* has been associated with seasonal peaks or temperature variation,^37^ and in our setting infection was more common during the wet season. This is important information especially for designing prevention and surveillance programs.

The traditional approach to diagnose *G. duodenalis* in low-resource countries is based on the detection of cysts in stool samples by direct microscopy. The method we used in this study (quantitative PCR) is both highly specific and sensitive^38^ and is likely to reflect the true magnitude of the infection compared with previous studies in Uganda. Nonetheless, we did not undertake further molecular typing, which would have clarified transmission pathways in this setting. We relied on the mother’s recall of the symptoms and their duration. The information collected could have been corroborated by recall bias. Because this was a cross-sectional study, we can report associations but not causality.

## CONCLUSION

Infection with *G. duodenalis* occurs in 1 of 15 children aged 9–36 months presenting to hospital with diarrhea or cough in Uganda. Overall, infection is associated with increasing age, use of and unimproved toilet facility, and wet season, whereas recurrent diarrhea, passing mucoid stool, and having a ruminant in the homestead are factors associated with *G. duodenalis* infection in children with diarrhea as a key symptom. Further studies are needed to clarify the zoonotic significance of *G. duodenalis* in this setting, with prevention strategies emphasizing personal protection measures in this age group especially during the wet season.
